# Circular RNAs—New Kids on the Block in Cancer Pathophysiology and Management

**DOI:** 10.3390/cells12040552

**Published:** 2023-02-08

**Authors:** Adrian Szczepaniak, Agnieszka Bronisz, Jakub Godlewski

**Affiliations:** 1 NeuroOncology Laboratory, Mossakowski Medical Research Institute, Polish Academy of Sciences, 02-106 Warsaw, Poland; 2 Tumor Microenvironment Laboratory, Mossakowski Medical Research Institute, Polish Academy of Sciences, 02-106 Warsaw, Poland

**Keywords:** cancer, cancer stem-like cells, extracellular vesicles, non-coding RNA, microRNA, circular RNA

## Abstract

The ever-increasing number of cancer cases and persistently high mortality underlines the urgent need to acquire new perspectives for developing innovative therapeutic approaches. As the research on protein-coding genes brought significant yet only incremental progress in the development of anticancer therapy, much attention is now devoted to understanding the role of non-coding RNAs (ncRNAs) in various types of cancer. Recent years have brought about the awareness that ncRNAs recognized previously as “dark matter” are, in fact, key players in shaping cancer development. Moreover, breakthrough discoveries concerning the role of a new group of ncRNAs, circular RNAs, have evidenced their high importance in many diseases, including malignancies. Therefore, in the following review, we focus on the role of circular RNAs in cancer, particularly in cancer stem-like cells, summarize their mechanisms of action, and provide an overview of the state-of-the-art toolkits to study them.

## 1. Introduction

Cancer is undeniably a first-tier healthcare problem and the leading cause of death in developed countries. According to GLOBOCAN data, there were 19.3 million new cases and 10 million deaths due to cancer worldwide in 2020 [1]. Research efforts on multiple fronts aim to find bona fide therapeutic targets that could be successfully applied to cancer therapy. As the research on protein-coding genes brought about significant yet incremental progress in the development of anticancer therapy, diagnosis, and prognosis, much attention is devoted now to understanding the role of non-coding RNAs (ncRNAs), which emerge as equally important players in tumorigenesis. They are involved in the global regulation of cellular processes, and abundant studies have shown that they can be used as potential therapeutic targets and biomarkers in cancer [2]. Numerous classes of functional ncRNAs have been described, including microRNAs, long non-coding RNAs (lncRNAs), and, more recently, circular RNAs (circRNAs), distinguished by the presence of a sequence that does not exist in host DNA or linear RNA. A large body of evidence (~235,000 original articles in PubMed to date) suggests that ncRNAs participate in complex regulatory networks that modulate the expression of a vast spectrum of genes [3].

Since 2010, the development of RNA-seq technology has vastly broadened the understanding of the transcriptome, including the verification of circRNAs landscape (circRNAome). Recently, numerous high-throughput and high-quality analyses were carried out to establish the circRNAome signatures specific to cancer cells. Due to the distinct character of circRNAs and an increasing number of reports, in this review, we summarize the recent discoveries considering the expression and role of circRNAs in various types of cancer, demonstrating them as the major players in cancer development and progression.

## 2. Non-Coding RNA

As the genetic information encoded in the double helix structure of DNA is transferred to the protein via messenger RNA (mRNA), that class of RNA molecules became the focal point of research for a long time. At the same time, other ncRNAs were put on the back burner, considered merely as co-factors in protein synthesis or with limited biological significance. The 21st century has brought tremendous advances with elaborate yet cost-effective sequencing, allowing the discovery of many previously unexplored areas, including the diverse ncRNA landscape [4]. The ENCODE (Encyclopedia of DNA Elements) project, started in 2005, showed that as much as 80% of the human genome is transcribed into some form of non-protein-coding RNA [5].

The evolution of ncRNAs provides a meaningful context. In general, ncRNAs are less conserved than protein-coding transcripts due to lessened selective pressure in the absence of strict reading frames. Moreover, the evolutionary emergence of organismal complexity ran concurrently with the increase in the non-protein-coding genome [6]. A high percentage of protein-coding transcripts characterizes prokaryotic genomes. Conversely, as the complexity arose, a significant relative reduction of protein-coding transcripts can be observed in the Eukaryotes. The correlation thus implies that the relative fraction of protein-encoding transcripts decreases as organisms become more complex. Therefore, the consensus is that the expansion of ncRNA enabled the emergence of more sophisticated regulatory systems, allowing organismal complexity to flourish [5].

**Figure 1:**
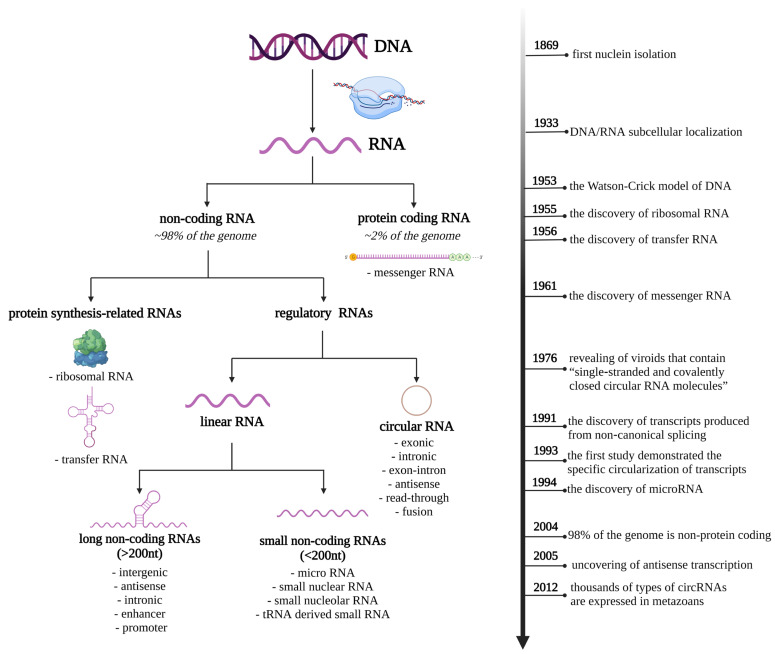
Classification of non-coding RNAs and the timeline of significant events discovering nucleic acids.

As ncRNAs are tightly regulated in various tissues, they emerge as crucial regulators of embryogenesis, differentiation, development, metabolism, and aging [7,8]. Moreover, as abundant ncRNAs were shown to play essential roles in physiological and pathological conditions, it has become apparent that they are intrinsically involved in various cancer-related processes. In addition, new functions of different groups of ncRNAs, such as circular RNAs, are still being discovered and described, indicating limited knowledge and the need to expand it. The classification of ncRNAs is based on various criteria (Figure 1), yet some difficulties in distinguishing categories persist due to their intersecting characteristics.

## 3. Non-Coding RNAs in Cancer

The expression of ncRNAs is cell-type- and tissue-specific and linked to the developmental stage and response to stressors or stimuli. Abundant studies have shown that ncRNAs are engaged in the genesis, and progression of various cancers, e.g., glioblastoma [9], leukemia [10], and carcinomas of the breast [11], liver [12], lung [13], skin [14], stomach [15], or colon [16].

ncRNAs can act at many stages of malignant transformation and tumor progression, ranging from boosting/suppressing the growth of primary tumors to promoting/inhibiting metastasis to distant organs. They can be, thus, broadly categorized as oncogenes and tumor suppressors in the same manner as their protein-coding relatives. Their mechanisms of action are diverse and involve adjustments to many signaling pathways, proteins, and RNAs. They often act in a context-specific manner (e.g., a particular cancer subtype) [17], providing targeting opportunities for treatment or precise diagnosis. Moreover, in addition to altering ncRNA-dependent pathways based on singular proteins and other RNAs, many ncRNAs can affect chromatin activity, thus altering entire transcriptomes and changing cell phenotypes at every level of their functioning. Thus, while ncRNAs play a crucial role in carcinogenesis, their action is often the sum of numerous minor alterations in particular genes/pathways activities or rely on genome-wide scale chromatin modifications and, as such, require more “big picture” approaches. Recent advances in research have brought to our attention the existence of circRNAs that seems to play a prominent yet poorly comprehended and likely underappreciated role in regulating cancer cell biology.

## 4. Circular RNA

Although circRNAs occur in all cells, they were initially thought to result from splicing errors. The first report on circRNAs appeared in 1976, describing viroids containing single-stranded and covalently closed circRNA molecules [18]. Fast forward to 2013, when thousands of circRNAs were identified in various mammalian systems, and it was the first time that it was suggested that some of them could modulate the activity of microRNAs [19]. This discovery opened a new research chapter focused on uncovering their role in the cell and the pathophysiology of many diseases. It is now part of the scientific consensus that circRNAs constitute an essential subpopulation of ncRNAs and are involved in the pathophysiology of many diseases. Among them are various cancers, neurodegeneration, cardiac fibrosis, and diabetes [20,21,22]. In addition to pathophysiological states, circRNAs play a significant role in organogenesis and differentiation. For example, hundreds of circRNAs are involved in the development of the heart and brain or the differentiation of cardiac precursors into cardiomyocytes.

**Figure 2:**
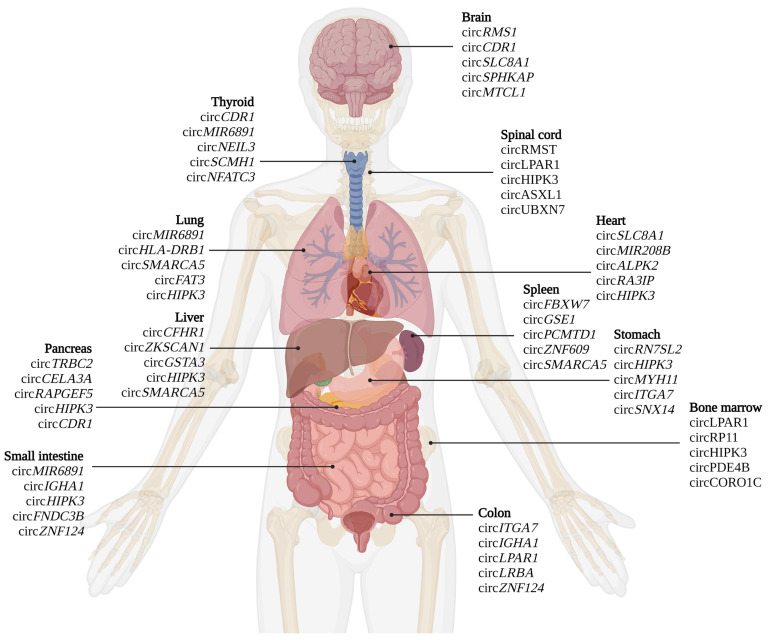
The top 5 circRNAs with the highest expression in a particular organ, according to the circAtlas database.

A specific expression profile of circRNAs characterizes each tissue, implying that their expression is tightly regulated and tailored to the function of the tissue/cell. However, circRNAs ubiquitously expressed in multiple tissues are also present. The pool of circRNAs between different tissues also varies. In the case of cancer, it has been identified that the highest number of condition-specific circRNAs is generated in brain tumors, while in skin cancer, the amount of such circRNAs is negligible [23]. We have presented the top 5 expressed circRNAs in a particular organ, according to the circAtlas database (Figure 2).

The most distinguishable feature of circRNAs among a plethora of ncRNAs is their unique structure. Generally, a single genomic location can generate different types of circRNAs. CircRNAs are generated by the mechanism dubbed back-splicing, in which the 3′ end of an exon binds to its 5′ end or an exon upstream through a 3′,5′-phosphodiester bond, forming a covalently closed-loop structure lacking 5ʹ to 3ʹ polarity [24]. Therefore, the lack of free ends makes them remarkably stable and resistant to exonucleases compared to their linear counterparts. The structure thus contributes to their stability and persistent presence in the cell—the average half-life of circRNAs was estimated at ~48 h, whereas mRNAs only ~10 h [25]. Thus, circRNAs, as molecules deregulated in many diseases and yet remarkably stable, are more promising candidates for therapeutic/diagnostic purposes than other ncRNAs.

To date, three hypothetical models of circRNA biogenesis mechanisms have been widely accepted: circularization mediated by RNA-binding proteins (RBPs) through regulation of neighboring splicing sites [26]; intron pairing-driven circularization, with flanking pre-mRNA introns containing inverted largely complementary sequences (e.g., GU-rich motifs and C-rich elements) thus enabling complementary pairing of both sides [27]; and lariat-driven circularization, in which complementary ALU flanking elements in intron regions compete with canonical splicing and accelerate circularization by reverse complementary matching [28].

Based on circAtlas database information—421,501 human circRNAs have been identified, with the brain as the organ with the highest number. However, with limited data, it is difficult to determine precisely how many circRNAs identified so far are associated with cancer. CircAtlas indicates that circRNAs derived from 284 host genes are associated with human cancer. However, this number is poised to change as functionally verified data accumulates.

The vast majority of circRNAs are transcribed from the nuclear genome. Although interestingly, circRNAs encoded by the mitochondrial genome have been recently discovered. They can facilitate the entry of nuclear genome-encoded proteins into mitochondria, resulting in mitochondrial rearrangements in such pathological conditions as cancer. For example, the mitochondrial genome-originating circRNA, mc-*COX2*, has been identified as positively associated with the progression of chronic lymphocytic leukemia [29]. However, the role of mitochondrial circRNAs remains elusive. Therefore, exploring their significance will expand the knowledge of circRNAs and may identify new therapeutic targets.

An increasing number of reports demonstrating novel functions of circRNAs has led to a more detailed categorization into four groups based on their biogenesis/structure: exon circRNAs, intron circular RNAs, exon-intron circRNAs, and intergenic circRNAs [30]. Members of each circRNA category are also characterized by their specific subcellular localization: exonic ones are mainly cytosolic, while other types, such as circular intron RNAs and exon-intron circRNAs, are enriched in the nucleus [31]. More recently, two other classes of circRNAs were described: fusion circRNAs (f-circRNAs) and read-through circRNAs (rt-circRNAs). F-circRNAs are commonly found in cancer cells and arise from chromosomal translocations or deletions, while rt-circRNAs result from read-through transcription [32], which occurs when the RNA polymerase starts transcription at the gene promoter, continues through the intergenic region, and terminates it beyond the “stop” codon, overlapping with a neighboring gene. Vo et al. identified over 1300 rt-circRNAs arising from the human genome in this way [23].

### 4.1. circRNA Databases

The last decade has brought significant advances in sequencing technologies, identifying almost innumerable mRNA splicing variants and non-polyadenylated RNAs, including circRNA.

CircRNAs can be identified in various ways. These include the analysis of sequencing records to determine the circularization site using multiple algorithms such as CIRI [33], Mapsplice [34], find_circ [35], CircRNAFisher [36], CIRCexplorer [37], or Acsf [38]. Databases/tools based on machine learning techniques can also predict circRNAs. Among them are PredcircRNA [39] (distinguishes circRNA from other lncRNAs), WebCircRNA [40] (an assessment of human genes and transcripts in terms of their potential to create circRNA), or DeepCirCode [41] (prediction of the circulation site for the formation of human circRNA).

Due to the increasing volume of data on circRNAs, we are witnessing the emergence of databases aiming to organize and expand the catalog of annotated circRNAs Me databases are designed explicitly for circRNAs. One such database is circBase [42], which contains such information as genome location, and circularization site read count data. Its latest version annotates circRNAs based on data derived from nine large-scale screening reports. Useful circRNA information can also be found at CircFunBase [43], CIRCpedia [44], CircRNADb [45], or CircBank [46]. Some databases aim to collect information about the whole ncRNA groups, allowing the analysis of the network of connections between different molecules. These include the CircInteractome database, which identifies RBP and microRNA binding sites on human circRNAs and provides tools to design siRNAs for circRNA silencing [47]. Another relevant example could be the CircNet database, which contains information about circRNA-microRNA-mRNA interaction networks [48]. We have summarized these key databases in Table 1, indicating their detailed functions.

### 4.2. circRNA Analysis Methods

Advances in high-throughput circRNA sequencing (circ-seq) analysis have identified thousands of circRNAs. Circ-seq allowing identification, annotation, and high-throughput screening has become the “gold standard” among methods [55]. In a complementary approach, microarrays are valuable tools for high-throughput analysis of the expression of specific circRNAs. Microarray probes are fixed on a solid support and explicitly designed to recognize circularization sites. Although, a downside aspect of microarrays is that they can profile only a limited number of known/predicted circRNAs [56,57].

Due to the uniqueness of circularization site sequence, a handful of molecular biology methods are used to identify/detect circRNAs, providing the ability to distinguish them from their linear counterparts. One is Northern blotting, employing probes that overlap either with a circularization site or a universal probe that recognizes both circular and linear transcripts from the same locus. RNase R digestion step is used in conjunction [58], thus removing linear RNAs while circular RNAs are enriched, allowing their further detailed analysis [59].

**Figure 3:**
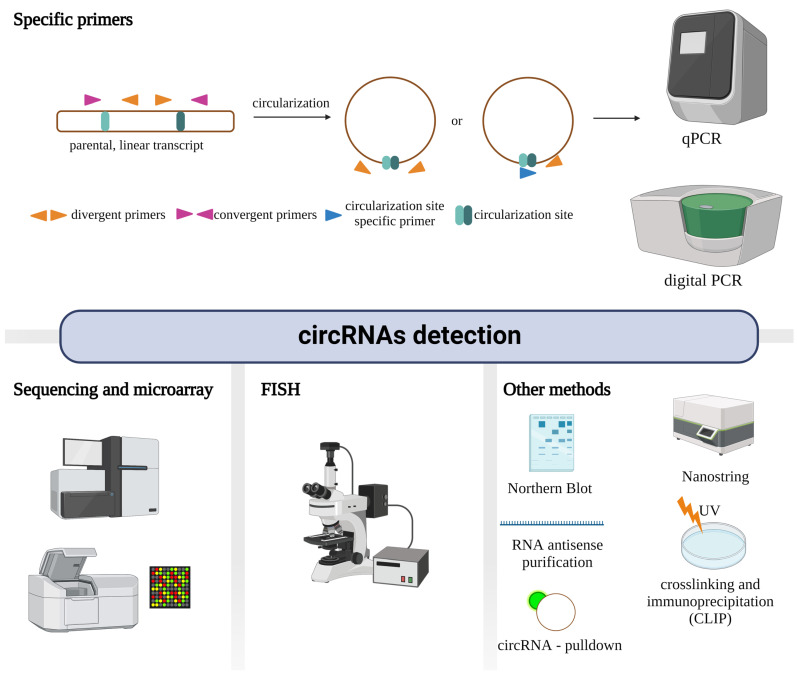
Summary of methods used to study circRNAs expression. The most commonly used methods for circRNAs detection are qPCR and digital PCR, which require primers (upper panel) that are specific for circRNAs’ unique sequence. Methods of global circRNA analysis, including sequencing, microarray, and Nanostring™ platform, are shown on the lower panel.

Quantitative PCR (qPCR)-based approaches are helpful when using a divergent primer pair designed to span the circularization site for specific amplification of circRNAs while disregarding the corresponding linear RNA (Figure 3). qPCR is routinely used to detect circRNAs and analyze reaction products by gel electrophoresis or sequencing [60]. In situ hybridization is also a helpful tool as a semi-quantitative measurement [61], providing additional information on the subcellular localization of selected circRNA. Digital PCR is regarded as a state-of-the-art technique as it yields more reproducible results and is highly sensitive, detecting even a single copy of a given transcript [62,63].

An essential element in discovering new biological functions of circRNAs is for delineating the extent of their relationships with proteins or DNA. The interactions of circRNAs with proteins can be studied using various methods classified as protein- or circRNA-centric, depending on the element from which the analysis starts. One of the most common methods is RNA immunoprecipitation (RIP). RIP uses a specific antibody directed against the protein of interest to isolate complexes of RBP and target RNAs [64]. Other complementary approaches include crosslinking and immunoprecipitation (CLIP) [65], fluorescence in situ hybridization (FISH) [66], or analysis of circRNA-ribosome association by sucrose gradient centrifugation [67]. Moreover, RNA antisense purification (RAP) coupled with mass spectrometry is a method that enables the identification of direct and specific protein interaction partners of a particular RNA molecule. RAP is also helpful in mapping RNA interactions with chromatin [31]. Thus, these methods allow mapping complex RNA-DNA-protein interactions to uncover intricate ncRNA networks.

Thus, as a new class of molecules, circRNAs require a new/adapted set of tools to profile their expression accurately and convincingly define their role (Figure 3).

### 4.3. Tools of circRNA Research

While various approaches have been developed to study the function of linear transcripts, the functional analysis of circRNAs remains challenging, requiring a unique research toolkit. As a result of a backsplicing-enforced circularization event, circRNAs possess a specific circularization site sequence, resulting in a characteristic exon-intron or exon-exon merging. This feature allows circRNA suppression by targeting the circularization site while leaving the linear parental transcript intact. It is noteworthy that circRNAs can arise from both protein-coding and non-coding transcripts.

**Figure 4:**
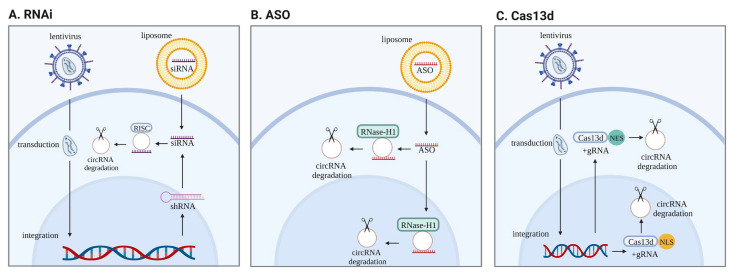
CircRNAs knockdown methods. (**A**)—RNAi consists of two approaches: permanent reduction of expression with shRNA or temporary with siRNA. A lentivirus with a plasmid containing shRNA integrates into the genome. In both methods, the functional molecule is a siRNA that binds complementarily to the target and causes its deterioration. (**B**)—ASO can act in both the cytosol and nucleus, and, by creating a DNA/RNA hybrid, leads to the degradation of a specific molecule via RNase-H1. (**C**)—A plasmid containing Cas13d and gRNA is delivered via a lentivirus into the cell and integrated into the genome. Cas13d can include either a NLS tag to localize in the nucleus or NES to localize mainly in the cytosol. Due to a specific gRNA, Cas13d attaches to a particular molecule and induces its degradation.

There are many approaches to altering the level of circRNAs in the cell. Among the most commonly used methods of RNA interference (RNAi)-based strategies are small interfering RNAs (siRNAs) and short hairpin RNAs (shRNAs) (Figure 4A). These approaches have also been relevant in modulating circRNA expression, yet the simultaneous silencing of cognate mRNAs significantly diminishes their applicability [68]. Therefore, other methods, such as antisense oligonucleotides (ASO), commonly used in protein and RNA research, are more advantageous in functional circRNA studies (Figure 4B). ASO are synthetic, single-strand RNA-DNA hybrids that reduce the expression of target transcripts via RNase H1. Chemically modified ASO are very stable and, thus, more practical than siRNA, especially in in vivo experiments.

Another tool that enables circRNA knockdown is CRISPR/Cas13d. Cas13d is an outlier method in the CRISPR world. The main feature distinguishing this new system from others is that Cas13d targets and cuts RNA instead of DNA. With Cas13d, the expression of circRNAs can be modulated regardless of subcellular localization (Figure 4C). Furthermore, Cas13d is a precise tool, revealing few, if any, off-targets compared to other RNA-based silencing methods [69]. The optimized gRNA design strategy for Cas13d allows for the knockdown of circRNA with an efficiency comparable to or higher than the widely used RNAi knockdown method [70].

Some circRNAs are expressed at low levels, so overexpression is required to verify their function. Overexpression of circRNAs can be achieved by introducing vector constructs with the circRNA sequence flanked by introns containing inverted repeat sequences, e.g., Alu repeats that hybridize and promote circRNA formation. The constructs can be introduced into the cell by plasmid transfection or viral infection [71].

## 5. Mechanism of Action of circRNAs in Cancer Cells

We have analyzed the publications available in PubMed. A search using the keywords “cancer” and “circular RNA” revealed 6514 publications, while the search for “circular RNA” and “cancer stem cells” revealed a record of 193 publications, as of January 2023. Cancer stem-like cells (CSCs) are a self-renewing, poorly differentiated, and highly tumorigenic subpopulation whose targeting is a critical step toward successfully eradicating the tumor. Therefore, here, we summarize the role of circRNAs in shaping stemness traits using several recent, relevant proof-of-concept examples.

**Figure 5:**
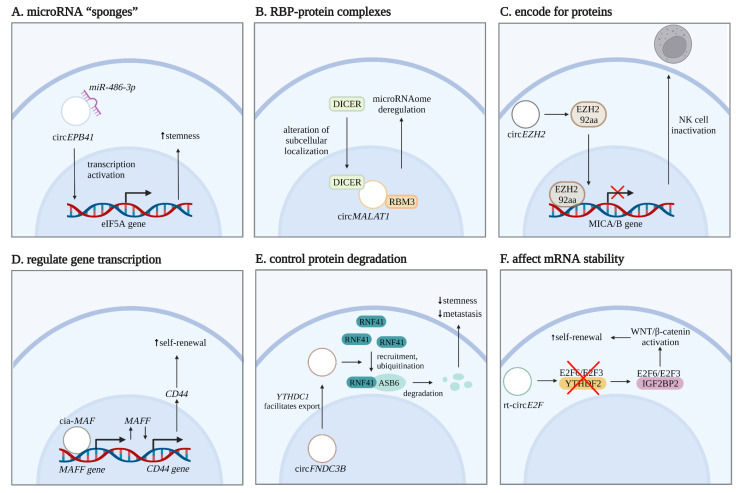
The mechanisms of action of circRNAs in cancer stem-like cells (based on recent reports). Circ*EBP41* acts as a sponge for *miR-486-3p*, effectively inducing the expression of eIF5A (*miR-486-3p* target) to maintain cell stemness (**A**). Circ*MALAT1* creates a nuclear RNA/protein complex with DICER and RBM3 proteins, thus, affecting the cell’s microRNAome composition (**B**). Circ*EZH2* encodes for EZH2-92aa peptide that inhibits NK cell activity (**C**). Cia-MAF (circRNA activating MAFF) binds to the MAFF promoter and enhances MAFF expression, which induces CD44 transcription, promoting stem cell traits (**D**). circ*FNDC3B* inhibits stemness and metastasis via RNF41-dependent ASB6 degradation (**E**). Rt-circ*E2F* promotes the association of *E2F6/E2F3* mRNAs with N-methyladenosine (m6A) reader IGF2BP2 and inhibits their association with another m6A reader, YTHDF2, thus promoting self-renewal of cancer stem-like cells (**F**).

CircRNAs were described to function in several ways, as shown (Figure 5):

- act as microRNA endogenous competitors known as “sponges” that mop up free, unbound microRNAs by presenting multiple microRNA target sites embedded within their sequence;
- form RBP-protein complexes or scaffolds for proteins—circRNAs can be involved in numerous processes by binding proteins as protein decoys or sponges;
- encode for proteins—the functions of short peptides encoded by circRNAs are generally on par with those of their full-length protein counterparts. Still, some of them seem to have differing functions [72,73];
- manipulate gene transcription by interacting with DNA or transcription factors;
- control protein degradation, e.g., through ubiquitination;
- regulate alternative splicing and affect mRNA stability.

### 5.1. circRNAs as microRNA Sponges

MicroRNAs are small, evolutionarily conserved ncRNA molecules whose genes are generously distributed across the human genome; approximately 2600 microRNAs have been identified [74]. They participate in post-transcriptional gene regulation, affecting such crucial cellular processes as proliferation, migration, metabolism, and differentiation. The microRNA genes are transcribed into primary microRNAs by RNA polymerase II and then processed by the DROSHA complex into pre-microRNAs exported to the cytoplasm and processed by DICER to produce mature microRNAs. They interact in the cytoplasm with the effector RNA-induced silencing complex (RISC), which binds their target mRNAs with a sequence complementary to the microRNA usually embedded within ‘mRNA’s 3′-UTR. They thus downregulate gene expression by either prompting mRNA degradation or blocking its translation at the ribosome [75].

Numerous circRNAs containing multiple complementary target sequences act as a sponge for specific microRNAs, thus effectively modulating the activity of entire signaling pathways. Unsurprisingly, many can modulate cellular protein functions by forming complex network interactions.

For example, circ*EPB41* (*hsa_circ_0000042*) promotes the stemness of non-small cell lung cancer cells in vivo and in vitro. Thorough analysis showed that *miR-486-3p* is a direct target of circ*EPB41*, and its expression was downregulated in tumorous tissues of non-small cell lung cancer patients. High-throughput sequencing and bioinformatics analysis showed that *miR-486-3p* targets the 3′UTR of eIF5A—a translation initiation factor that regulates tumor stem cell differentiation. Moreover, the quelling of circ*EPB41* suppressed self-renewal capacity and decreased the expression of stemness markers. Conversely, downregulation of *miR-486-3p* or overexpression of eIF5A restores cell proliferation and invasiveness after circ*EPB41* silencing [76].

Evidence suggests that microRNA-sponging circRNAs are also involved in regulating breast cancer CSCs’ function and invasion capacity. Liu et al. identified circ*NOLC1* as a competing endogenous RNA for *miR-365a-3p*, thus enhancing the expression of STAT3. Downregulation of circ*NOLC1* expression inhibited the ability of pleural effusion-derived breast cancer cells to form mammospheres and led to the suppression of stemness-related genes. Notably, the overexpression of STAT3 rescues circ*NOLC1* depletion-attenuated proliferation and CSC activity in breast cancer. The authors also indicated that the overexpression of circ*NOLC1* rescues propofol-attenuated proliferation of breast cancer CSCs [77].

Epstein-Barr virus (EBV)-associated gastric cancer is a distinct subtype of this malignancy with unique molecular characteristics [78], yet, until recently, it was unclear whether CSCs exist and play any role in its physiopathology. By long-term treatment of the EBV-associated gastric cell line with a chemotherapeutic agent in mice, a highly aggressive cell line was obtained and consisted mainly of stem-like CD44+/CD24-subpopulation. These cells expressed an EBV-encoded circRNA, ebv-circ*LMP2A*, that induces and maintains stemness phenotypes. To this effect, ebv-circ*LMP2A* sponges *miR-3908* and enhances the TRIM59/p53 pathway, and its high expression is significantly associated with metastasis and a poor prognosis in patients with this type of cancer. These findings have thus provided evidence for the existence of CSCs in EBV-associated gastric cancer, shedding light on the pathogenic mechanism of ebv-circ*LMP2A* [79].

Thus, circRNA-mediated microRNA sponging emerges as an emblematic competing endogenous RNA mechanism that enables forces significant enough to shape cancer cell phenotypes.

### 5.2. circRNAs as Components of Protein Complexes

The patterns of circRNA interaction with proteins are seemingly more complex than circRNA-microRNA interactions, as several different modes of action have been described. It has been confirmed that binding between circRNAs and proteins affects the subcellular localization of protein complexes and can alter the levels and longevity of both RNA and protein players [80,81,82].

The DICER protein complex is a critical factor in the biogenesis of most small regulatory RNAs. This enzyme belongs to the ribonuclease III family, which cleaves long double-stranded RNA molecules into short molecules, including microRNAs and siRNAs. DICER malfunction is associated with the global loss of mature microRNAome in both in vitro and in vivo models, supporting pro-oncogenic cellular transformation [83]. MicroRNAome, an assortment of all microRNAs being expressed in a given cell at a given time, is an essential readout of cellular homeostasis. Faulty expression or localization of microRNA processing machinery components leads to impaired microRNAome composition and is thought to be associated with cancer formation [80]. However, the mechanism of such deregulation remained unclear. A comparison of DICER distribution in the cellular compartments of glioblastoma CSCs and non-malignant neural progenitor cells (NPCs) indicated that while DICER was mainly cytosolic in NPCs, it was decidedly nuclear in various glioblastoma CSC subtypes. Detailed studies discovered a nuclear RNA/protein complex consisting of the DICER, strictly nuclear RBM3 protein, and circ*MALAT1* (*hsa_circ_0002082*) derived from the long non-coding *MALAT1* oncogene. Knockdown of circ*MALAT1* restored cytosolic DICER localization, thus reestablishing microRNAome homeostasis in diverse subpopulations of glioblastoma CSCs. The significance of circ*MALAT1* was apparent upon its knockdown in glioblastoma CSCs, resulting in lessened clonality and tumorigenicity and prolonged survival of circ*MALAT1* knockdown glioblastoma CSC tumor-bearing animals [80].

Due to their ability to fine-tune scores of genes, microRNAs have been recognized as master guardians of final differentiation. Most microRNAs dictate terminal differentiation programs; therefore, the molecular signature of mass inhibition of microRNAs allows cancer cells to avoid these programs [84]. The discovery of sweeping deregulation of the microRNAome in glioblastoma CSCs (via circ*MALAT1* action) may indicate a potential therapeutic target for unleashing differentiation programs in these cells. Restoring the pre-malignant composition of the microRNAome promotes the differentiation of cancer stem-like cells, making them more susceptible to treatment. Such an approach would thus allow the eradication of a subpopulation vital for tumor recurrence post-therapy.

CircRNAs can also modulate the activity of the transcription factors. The circRNA circ*RPPH1* (*hsa_circ_0000512*) is elevated in glioblastoma CSCs, correlating with poor patient survival. It binds with UPF1 (RNA helicase), which maintains the stability of a complex, and ATF3 (transcription factor), which increases *UPF1* transcription and activates TGF-β signaling. Significantly, the characterized feedback loop contributes to the constant expression of the stem cell marker—Nestin, maintaining the oncogenic features of glioblastoma CSCs. The silencing of circ*RPPH1* significantly inhibited the proliferation and clonogenicity of glioblastoma CSCs both in vitro and in vivo, while its overexpression enhanced their self-renewal [85].

CircRNAs were also implied in liver carcinoma CSCs, where circRNAs’ role has so far remained elusive. Recently, circ*IPO11* was shown to be significantly increased in these CSCs, where it recruited topoisomerase 1 to the *GLI1* gene promoter, leading to the activation of Hedgehog signaling that plays a critical role in the self-renewal of liver cancer CSCs’ tumorigenicity [86].

In summary, circRNAs can modulate the expression/activity of specific protein complexes by affecting the sub-cellular localization of essential components, disrupting complex assembly, turnover, composition, and activity, thus affecting entire pathways and complex molecular readouts.

### 5.3. circRNAs Encode Proteins

Linear transcripts that are the matrix for protein synthesis contain certain structural elements, e.g., the 5′ cap structure, required for translation. CircRNAs, as molecules lacking these structures, were thus regarded as true non-protein-coding RNAs. However, studies have shown that specific and arguably rare circRNAs can serve as a template for protein synthesis. Some circRNAs, especially exonic ones, contain open reading frames (ORFs) that can be actively translated [87].

Due to their unique structure, circRNAs are thought to be translated primarily through cap-independent mechanisms such as through an internal ribosome entry site (IRES) [88] and N6-methyladenosine (m6A) modification [89], although the mechanisms of translation of many coding circRNAs are still unknown.

Emerging evidence suggests that circRNA-derived proteins play a significant biological role in the cellular stress response and are involved in cancer progression. A representative example of this mechanism of action is recently described as *hsa_circ_0006401*, whose expression in metastatic colorectal cancer was significantly increased compared with non-metastatic one [90]. This particular circRNA contains an ORF spanning a splicing junction that encodes a 198 amino acid-long peptide expressed in human colon cancer and adenocarcinoma tissue samples, promoting an aggressive phenotype in colorectal cancer cells [90].

CircRNAs, which encode proteins, have also been described in CSCs. A recent study indicated that the circular E-cadherin, which encodes a previously unknown peptide variant of the secretory E-cadherin protein, promotes the tumorigenicity of glioblastoma CSCs. E-cadherin protein activates EGFR independently of EGF, while its inhibition significantly suppresses tumorigenicity [91]. In another case, a protein encoded by circ*EZH2*—EZH2-92aa, overexpressed in glioblastoma cells, induced the evasion of glioblastoma CSCs’ responses to NK cells. EZH2-92aa inhibits the transcription of Major Histocompatibility Complex class I polypeptide-related sequence A/B and indirectly inhibits the transcription of UL16-binding protein by stabilizing EZH2. A functional approach showed that stable knockdown of EZH2-92aa enhances NK cell-mediated glioblastoma CSCs eradication in vitro and in vivo, synergizing with anti-PD1 therapy. Thus, the EZH2-92aa peptide encoded by circ*EZH2* is a decisive immunosuppressive factor [92].

The translation potential Is a previously underappreciated and exciting line of research in the context of circular RNAs. The discovery of their coding capabilities shed new light on their function and unexpectedly shattered their reputation as true non-coding molecules. In determining the function of proteins derived from circRNAs, it should be noted that some have functions that mirror their host genes’ products, while some may have roles that are at odds with the parental transcripts’ products. Many questions remain open despite discovering potential mechanisms of circRNAs translation and identifying many functional peptides encoded by these non-coding RNAs. It is thus vital to delineate the biological significance of circRNAs translation and determine the translation mechanisms that enable protein synthesis from circRNAs.

### 5.4. circRNAs Regulate Gene Expression

Some circRNAs regulate gene expression at the post-transcriptional stages, e.g., by acting as molecular sponges that bind to and block microRNAs. Others can regulate the expression of their parental genes via attracting transcription factors and chromatin modifiers. Recently, a circRNA called circRNA activating *MAFF* (cia-*MAF*) was identified. It is highly expressed in liver cancer and its CSCs. Cia-*MAF* binds to the *MAFF* promoter, recruits the TIP60 chromatin-modifying complex, and ultimately promotes *MAFF* expression. As a result, *MAFF* promotes the expression of *CD44*, a crucial CSCs’ marker that upholds their self-renewal. Loss of cia-*MAF* function weakens the link between the TIP60 complex and the MAFF promoter [93]. In another study, downregulation of circ*REEP3* (*hsa_circRNA_400564*) inhibits the tumorigenicity of colorectal cancer and its metastatic potential while impairing their stemness. Mechanistically, circ*REEP3* recruits the chromatin-remodeling protein CHD7 to the promoter of the renowned oncogene FKBP10, activating its transcription. Additionally, circ*REEP3* enhanced the interaction between RIG-1 and RNF125 to promote ubiquitination-dependent degradation of RIG-1, leading to the suppression of antitumor immunity [94].

Thus, circRNAs can orchestrate the interaction of transcription factors with promoters by recruiting proteins or entire complexes, consequently shaping the gene expression landscape in CSCs.

### 5.5. circRNAs Control Protein Lifespan and Turnover

CircRNAs can regulate the pool of proteins present in a cell at any given time by controlling their lifespan and possible degradation. One example of such a circRNA is circ*FNDC3B* (*hsa_circ_0006156*). m6A-modified circ*FNDC3B* plays a tumor suppressive role in colon cancer CSCs by increasing *RNF41* mRNA stability and expression and thus promoting ASB6 degradation via RNF41-mediated ubiquitination. RNF41 silencing abrogated circ*FNDC3B*-suppressed stemness and metastatic potential of colorectal CSCs. In vivo experiments showed that overexpression of circ*FNDC3B* or RNF41 alone suppressed tumor growth, stemness, and liver metastasis through modulation of ASB6 [95].

Although many studies have covered the detailed mechanistic and signaling contexts of the ferroptosis pathway, the role of ncRNAs, especially circRNAs, in the process is still unclear. One such molecule is circ*LRFN5* (*hsa_circ_0031751*), which modulates ferroptosis in glioblastoma CSCs. This circRNA is downregulated in glioblastoma compared to normal brain tissues. It has been shown that circ*LRFN5* can inhibit glioblastoma CSCs’ viability, neurosphere formation, stemness, and tumor formation in vivo by binding to the PRRX2 protein, thus promoting its degradation via a proteasomal pathway mediated by ubiquitin. As PRRX2 maintains the expression of GTP cyclohydrolase I (GCH1), its degradation disables GCH1 activity, leading to lipid and ROS accumulation as well as glutathione depletion, thereby inducing ferroptosis and reducing the carcinogenicity of CSCs [96].

In conclusion, circRNAs regulate protein stability in CSCs by inhibiting proteins’ activity, stability, and turnover, thus participating in a dense network of connections, e.g., regulating cell death.

### 5.6. circRNAs Regulate mRNA Stability

CircRNAs can also manage the pool of mRNAs present in the cell and their biological activity. Chen et al. identified an rt-circRNA, rt-circ*E2F*, which is highly expressed in liver cancer and CSCs and plays an essential role in their self-renewal and activity. Rt-circ*E2F* interacts with *E2F6* and *E2F3* mRNAs, attenuating their turnover, thus, increasing *E2F6/E2F3* activity. Moreover, this circRNA promotes the association of *E2F6/E2F3* mRNAs with IGF2BP2 and inhibits their association with m6A reader—YTHDF2, thereby inhibiting *E2F6/E2F3* mRNA decay. Both *E2F6* and *E2F3* are required for the self-renewal of liver CSCs and activation of the Wnt/β-catenin pathway. Thus, inhibiting these pathways is a promising strategy for preventing liver tumorigenesis and metastasis [97]. 

Although the number of reports on such functions of circRNAs in CSCs is limited, recent studies confirm that circRNAs can affect RNA turnover in this type of cells.

## 6. Extracellular Vesicle-Derived circRNAs in Cancers

Intercellular signaling, a fundamental process of receiving and transmitting signals to and from the surrounding microenvironment, is often mediated through the exchange of extracellular vesicles (EVs), a heterogeneous group of membrane structures shed by cells. Cancer cells derived EVs promote cell growth and survival, shape the tumor microenvironment (TME), enhance invasive capacity, and induce therapy resistance [98] carrying diverse cargo that includes peptides, ncRNAs, mRNAs, and DNA fragments [99].

The analysis of EV content can be a handy tool for the diagnosis, prognosis, and measuring of the response to the different treatment regimens. Due to their extended half-life and high specificity of detection, circRNAs constitute promising biomarker candidate molecules. Stella et al. described circ*SMARCA5* (*hsa_circ_0001445*) and circ*HIPK3* (*hsa_circ_0000284*) as potential glioblastoma biomarkers localized in serum extracellular vesicles (sEVs). Both circRNAs were significantly less abundant in sEV from high-grade glioblastoma patients than in healthy individuals and patients with less advanced stages of the disease. An analysis based on the expression of circ*SMARCA5* and circ*HIPK3* derived from sEV allowed the distinguishing of glioblastoma patients from healthy controls with high accuracy [100]. Hon et al. identified 105 significantly up-regulated and 34 down-regulated circRNAs in EVs secreted by therapy-resistant colorectal cancer cells and selected EV *hsa_circ_0000338* as a potential biomarker in colorectal cancer [101].

The authors of a study published in 2021 developed a classifier of circRNA extracted from urine-derived EVs that could detect high-grade prostate cancer. They also developed a reproducible and non-invasive tool called Ccirc that showed higher accuracy than two standard risk calculators in several patient cohorts [102]. Thus, it becomes a consensus that EV-encapsulated circRNAs secreted by tumor cells are poised to deliver new diagnostic biomarkers.

Importantly, EVs and their cargo can be used in cancer therapy. In a recent report, bone marrow mesenchymal stem cells (BM-MSCs), whose EVs contained *hsa_circ_0030167*, were used to assess tumor-suppressive properties. It has become apparent that such BM-MSC-derived EVs significantly reduced the invasiveness and stemness of pancreatic cancer cells with *hsa_circ_0030167* by sponging *miR-338-5p*, enhancing the expression of Wif1 and consequently inhibiting the Wnt8/β-catenin pathway. Moreover, in a mouse model, pancreatic tumors exposed to such BM-MSC-derived EVs were significantly smaller, suggesting that they inhibit tumor progression in vivo [103].

To date, evidence has been provided that circRNAs derived from Evs affect tumor metabolism, mediate tumor metastasis, induce cell migration, or modulate drug resistance. Although considerable progress has been made in studying the role of those circRNAs, information linking basic research observations with a clinical application for diagnostic and prognostic purposes is still lacking [104].

## 7. Role of Circular RNAs in Tumor Immune Microenvironment

The TME is considered the “soil” for tumor cell growth in both primary and metastatic sites. TME consists of a cellular compartment—not only tumor cells but also immune cells and those making up the stroma, along with an extracellular matrix and numerous signaling molecules. It is important to emphasize that the dynamic flow of signals between the tumor and the surrounding microenvironment significantly impacts tumor initiation, development, and response to therapy [105].

Due to the growing interest in cancer immunotherapy, much attention is devoted to describing and modifying the tumor immune microenvironment (TIME) in an immunotherapeutic context. Efforts to intensify the immune response and eliminate cancer cells have led to the development of immunotherapy-boosting approaches in the TIME context that include checkpoint blockade, CAR-T cells, cancer vaccines, and oncolytic virus therapy [106].

CircRNAs can regulate the activity/localization of different groups of immune cells in TIME. For example, high expression of circ*ARSP91* (*hsa_circ_0085154*) [84] induces NK cell cytotoxicity against liver cancer cells. In contrast, EVs secreted by hepatocellular carcinoma cells contain circ*UHRF1* (*hsa_circ_0048677*) that inhibits the secretion of IFN-γ and TNF-α by NK cells via the degradation of *miR-449c-5p* [107]. Tumor-infiltrating lymphocytes are immune cells capable of highly specific immune reactivity; significantly, their higher percentage correlates with a better prognosis [108]. In recurrent nasopharyngeal carcinoma, circ*0000831*, circ*0006935*, circ*005019*, circ*0031584*, and circ*0001730* affect the distribution of immune cells and decrease the ratio of CD4+/CD8+ T cells [109]. A balance between M1 macrophages (responsible for the onset of inflammation) and M2 macrophages (have anti-inflammatory effects) is modified by the circRNA landscape. Zhang et al. showed that circ*003780*, circ*010056*, and circ*010231* were enriched in M1 cells, whereas the expression levels of circ*003424*, circ*013630*, circ*001489*, and circ*018127* were downregulated in M1 macrophages [110].

Thus, evidence accumulates to support the role of circRNAs in modulating TIME, contributing to modified immunotherapy’s effectiveness. However, the data are still scarce, and further efforts are necessary to achieve a fuller, more conclusive picture.

## 8. Role of circRNA Stoichiometry in Their Action

For circRNAs to achieve the efficient biological activity, their stoichiometry must be appropriate and tailored to the specific case, such as the abundance of the targeted microRNA. A stoichiometric balance between targets and circRNAs must be maintained to accomplish this. CircRNAs may be ejected from the cell via EVs, thus affecting their functionality [94]. Moreover, a single gene can generate multiple circRNAs, with a variable number of copies or different sizes (e.g., the *PTK2* gene can produce 47 different circRNAs). All this affects the result—biological effect or lack thereof [94].

RNA modifications, such as m6A, m1A, m5C, and pseudouridine, mold the epitranscriptome. Changing the stoichiometry of modifications can generate functional diversity in the RNA transcripts. One of the most abundant RNA modifications is m6A, which was demonstrated to play a role in the cap-independent initiation of circRNA translation. Therefore, epitranscriptomic modifications also affect the stoichiometry of biologically active circRNAs or their peptide products.

## 9. Circular RNAs in Cancer Stem-like Cells Differentiation

CSCs are a severe obstacle to successful therapy, so the quest for approaches toward implementing cellular differentiation programs is currently in the research crosshairs. Lu et al. summarized the role of circRNAs in normal stem cells and noted that their final differentiation could be induced by modifying the expression of individual circRNAs [111]. Some recent studies have also described the role of circRNAs in differentiating CSCs, yet these reports are few. For example, Jiang et al. identified circ*MEG3*, which is downregulated in liver CSCs negatively correlates with telomerase expression. Circ*MEG3* inhibits telomerase activity and shortens HULC- and Cbf5-dependent telomere lifespan. Remarkably, increased Cbf5-telomerase activity abolishes the ability of circ*MEG3* to inhibit the malignant differentiation of liver CSCs [112]. Although, more studies are needed to describe the potential role of circRNAs in the forced differentiation of CSCs, to support the trend of current research.

## 10. CircRNAs in Cancer Management

A robust biomarker should be stable and site- or condition-specific in detection. CircRNAs are thus poised to be valuable cancer biomarkers due to their exceptionally high stability, unique sequence, and prevalence in bodily fluids, e.g., plasma. Consequently, the analysis of the circRNA profiles becomes an indispensable aspect of gene expression analysis. Thus, not only perturbations in the expression of coding genes but also of circRNAs can serve as indices of a cell’s state and fate [113,114].

CircRNA molecules are sought after not only as biomarkers but also as potential therapeutic targets. As we outlined above, multiple studies demonstrated benefits from modulating the expression of circRNAs. The unique circularization sequence is a considerable asset, allowing precise modification of their expression. One of them is the use of ASOs, which were demonstrated to be effective in numerous clinical trials and approved for treatment in some conditions [115,116,117]. Based on the NIH’s website (ClinicalTrials.gov, (accessed on 27 December 2022), there are currently seven clinical trials aimed at pinpointing circRNAs as biomarkers or therapeutic candidates in various types of cancer. Unquestionably, we currently witness a great quest for further studies, including in vivo and preclinical studies, to confirm circRNAs’ utility/efficacy in the clinic.

## 11. Conclusions

Cancer remains a universal health problem despite years of extensive research. A growing volume of evidence has consistently confirmed the importance of the new class of ncRNAs—circRNAs—molecules affecting cancer development. CircRNAs act as microRNA sponges and form complexes with proteins within vital tumor-suppressive and pro-oncogenic hubs such as p53, VEGF, or c-myc to regulate the expression of gene networks and encode peptides in various types of cancer cells [81,118,119,120]. Most of their modes of action discovered so far implicate them as modifiers of the action of individual RNAs. Still, it becomes increasingly apparent that they can also act globally. Moreover, circRNAs are considered novel and promising candidates for ‘pathologies’ biomarkers due to their high stability and specificity in detection by the unique circularization site.

The mechanisms of circularization are still not fully understood. An apparent gap in our knowledge exists concerning the cellular mechanisms/signals that induce and inhibit the formation of circRNAs based on linear transcripts in cells. The relevant readout would be a ratio between circular and linear transcripts originating from the same locus. Determining this balance (and the mechanisms behind it) in different pathological states would be a tempting approach to linking the ratio of these two transcripts with, for example, cell tumorigenicity. Instead of targeting an already formed circRNA, potentially, it could be beneficial to disrupt the process of its formation.

CSCs exhibit both self-renewal and multi-lineage differentiation capabilities. They are associated with tumor recurrence, and their eradication is necessary for effective treatment. As some circRNAs promote stem-like traits, their targeting can considerably affect CSCs’ emergence and vitality. As we have described, distorted molecular readouts, e.g., the microRNAome, play an essential role in promoting stemness. Current understanding does not fully grasp the mechanisms of microRNAome suppression during tumorigenesis and how vital microRNA homeostasis is in tipping the balance between differentiation and stemness. Importantly, circRNAs emerge as guardians of the microRNAome, e.g., circ*MALAT1*, which disrupts the microRNA maturation machinery [81]. Based on this example, the possibility of discovering an exciting relationship arises, showing that a single circRNA can create a ripple effect that disrupts the function of multiple effectors in a cell. Delivery of several singled-out microRNAs to cells to inhibit cancer progression has shown only limited efficacy [121,122]. Thus, an innovative approach would be to restore the homeostasis of thousands of molecules or whole molecular readouts by modulating the expression of a single specific agent.

Looking at the importance of circRNAs from another point of view, i.e., taking into account their formidable numbers, it is unlikely that most circRNAs have direct functions; the contrary might be true. While an altered circRNA profile is unlikely to indicate causal involvement in disease, it may indicate abnormal transcription or splicing of the parental gene or missteps in RNA editing. A careful analysis of the circRNA landscape can therefore indicate factors contributing to disease, even though many circRNAs may not be functional but represent transcriptional malfunction.

It is also plausible that the transformed cell can promote the circularization by yet-undetermined mechanisms, directing its transcriptional output toward increased circularization of specific transcripts or the overall enhancement of its circRNAome.

CircRNAs are a newly emerging and exciting field of cancer research. Determining the composition of the circRNAome, localizing individual circRNAs, and establishing their functions by deconstructing their dense network with protein complexes and other RNA species increasingly seems similar to a bona fide research goal. The discovery and more granular delineation of the circRNAs’ footprint would add a new, more comprehensive dimension to the research of the RNA universe and provide novel mechanistic insight and avenues for therapeutic intervention.

## Figures and Tables

**Table 1:** Databases commonly used for the analysis of circRNAs.

**Database**	**Resources**	**Options**	**Link**	**Ref.**
CircBase	public circRNA datasets.	Sequence-based search; Search the database by identifier, gene description, and genomic position; Retrieve dataset slices by defining a set of conditions (table browser); Export FASTA files containing the genomic sequence.	http://circbase.org (accessed on 29 December 2022)	[42]
CircFunBase	7059 functional circRNAs entries from 15 organisms, including 7 plants and 8 animals.	Extracting such information as circRNA name, position, tissue, expression pattern, detection tool, function, gene symbol, gene description, PubMed ID, GO annotations, and circRNA-associated miRNAs; Visualization of miRNA-circRNA and RBP-circRNA interactions.	http://bis.zju.edu.cn/CircFunBase/ (accessed on 29 December 2022)	[43]
CIRCpedia	circRNA annotations from over 180 RNA-seq datasets across six different species.	Search, browse, and download circRNAs with expression characteristics/features in various cell types/tissues; Conservation analysis of circRNAs between humans and mice; Comparison of circRNA expression between samples.	http://www.picb.ac.cn/rnomics/circpedia (accessed on 29 December 2022)	[44]
CircRNADb	32,914 human exonic circRNAs selected from diversified sources.	Data search, browse, download, submit and feedback for the user to study particular circular RNA of interest; Genomic information, exon splicing, genome sequence, internal ribosome entry site (IRES).	http://reprod.njmu.edu.cn/circrnadb (accessed on 29 December 2022)	[45]
CircBank	140,790 human circRNAs.	Extracting such information as: miRNAs binding sites; Conservation across species; m^6^A modifications; Mutation in circRNAs; Protein-coding potential; Predicted IRES sites.	www.circbank.cn (accessed on 29 December 2022)	[46]
CircInteractome	public circRNA, miRNA, and RBP databases.	Searches circRNAs name; Searches the genomic position and best-matching transcripts of the circRNA; Retrieves genomic and mature circRNA sequences; Searches RBPs binding to a circRNA and sequences upstream/downstream of the circRNA; Identifies RBPs binding to the circRNA junctions; Identifies miRNAs targeting a circRNA; Designs divergent primers for circRNAs; Designs siRNAs Specific to circRNA.	https://circinteractome.nia.nih.gov (accessed on 29 December 2022)	[47]
CircNet	2732 samples from 37 types of cancers.	Detect circRNA; Construct full-length sequence; Calculate circRNA expression; circRNA-miRNA interaction; miRNA-gene interaction; Network construction.	https://awi.cuhk.edu.cn/∼CircNet (accessed on 29 December 2022)	[49]
CircAtlas	1070 RNA-seq samples collected from 19 normal tissues across six vertebrate species; 1,007,087 highly reliable circRNAs.	Exploring circRNA-miRNA and RBP-binding sites; Identification of the orthologous genes expressing orthologous circRNAs; circRNA detection and full-length transcript construction;	http://159.226.67.237:8080/new/index.php (accessed on 29 December 2022)	[50]
CircMine	1 821 448 entries formed by: 136 871 circRNAs; 87 diseases; 120 circRNA transcriptome datasets of 1107 samples across 31 human body sites.	circRNA-miRNA prediction; circRNA IRES prediction; Group samples based on their clinical metadata and setting parameters for individual analysis.	http://www.biomedical-web.com/circmine/ (accessed on 29 December 2022)orhttp://hpcc.siat.ac.cn/circmine/home (accessed on 29 December 2022)	[51]
StarBase	108 CLIP-Seq data from 37 studies.	Decodes the Interaction Networks of lncRNAs, miRNAs, competing endogenous RNAs, RNA-binding proteins (RBPs), and mRNAs from large-scale data.	https://starbase.sysu.edu.cn/starbase2/index.php (accessed on 29 December 2022)	[52]
Circad	1388 disease-related circRNAs.	Provides standard disease nomenclature as per the ICD codes; It additionally lists the assay and PCR primer details, including experimentally validated ones, as a ready reference for researchers.	https://clingen.igib.res.in/circad/index.html (accessed on 29 December 2022)	[53]
Circ2Disease	5368 associations; 237 circRNAs; 217 diseases; 313 miRNAs sponges; 1746 miRNA targets; 647 RBPs.	Browse the experimentally supported circRNA-disease association data; Search associations by particular circRNA or/and disease name or other keywords; Download all experimentally supported circRNA-disease association data; View circRNA–miRNA-gene regulatory networks in human diseases.	http://bioinformatics.zju.edu.cn/Circ2Disease/index.html (accessed on 29 December 2022)	[54]

## Data Availability

Data sharing not applicable. No new data were created or analyzed in this study. Data sharing is not applicable to this article.

## Abbreviations

ASO	antisense oligonucleotide
BM-MSCs	bone marrow mesenchymal stem cells
circRNA	circular RNA
CLIP	crosslinking and immunoprecipitation
EBV	Epstein-Barr virus
EVs	extracellular vesicles
FISH	fluorescence in situ hybridization
f-circRNAs	fusion circRNAs
gRNA	guide RNA
HIF-1α	hypoxia-inducible factor-1α
lncRNA	long non-coding RNA
mRNA	messenger RNA
ncRNAs	non-coding RNAs
NPCs	neural progenitor cells
RAP	RNA antisense purification
RBP	RNA-binding protein
RIP	RNA Immunoprecipitation
RISC	RNA-induced silencing complex
RNAi	RNA interference
rt-circRNAs	read-through circRNAs
sEVs	serum extracellular vesicles
shRNAs	short hairpin RNAs
siRNA	small interfering RNA
TIME	tumor immune microenvironment
TME	tumor microenvironment
